# Risk Factors for the Presence of Anal Intraepithelial Neoplasia in HIV+ Men Who Have Sex with Men

**DOI:** 10.1371/journal.pone.0084030

**Published:** 2013-12-18

**Authors:** Olivier Richel, Henry J. C. De Vries, Marcel G. W. Dijkgraaf, Carel J. M. Van Noesel, Jan M. Prins

**Affiliations:** 1 Department of Internal Medicine, Division of Infectious Diseases, Academic Medical Center, Amsterdam, The Netherlands; 2 Department of Dermatology, Academic Medical Center, Amsterdam, The Netherlands; 3 Clinical Research Unit, Academic Medical Center, Amsterdam, The Netherlands; 4 Department of Pathology, Academic Medical Center, Amsterdam, The Netherlands; 5 Centre for Infection and Immunity Amsterdam (CINIMA), Academic Medical Center, Amsterdam, The Netherlands; 6 STI outpatient clinic, Cluster for Infectious Diseases, Public Health Service Amsterdam, Amsterdam, The Netherlands; Public Health Agency of Barcelona, Spain

## Abstract

**Objective:**

Anal Intraepithelial Neoplasia (AIN) is present in the majority of HIV+ men who have sex with men (MSM) and routine AIN-screening is subject of discussion. In this study we analysed a wide range of potential risk factors for AIN in order to target screening programs.

**Methods:**

We screened 311 HIV+ MSM by high resolution anoscopy, with biopsies of suspect lesions. HIV-parameters, previous sexual transmitted infections (STI’s), anal pathology, sexual practices and substance use were analysed in relation to AIN by uni- and multivariable logistic regression.

**Results:**

AIN (any grade) was found in 175/311 MSM (56%), high grade (HG)AIN in 30%. In the univariable analysis, years since HIV diagnosis, years of antiretroviral therapy (cART) and anal XTC use decreased AIN risk, while a history of anogenital warts and use of GHB (γ-hydroxybutyric acid) increased this risk. In the multivariable analysis three parameters remained significant: years of cART (OR=0.92 per year, p=0.003), anal XTC use (OR=0.10, p=0.002) and GHB use (OR=2.60, p=0.003). No parameters were significantly associated with HGAIN, but there was a trend towards increased risk with anal enema use prior to sex (>50 times ever; p=0.07) and with a history of AIN (p=0.06). CD4 count, STI’s, anal pathology, smoking, number of sex partners and anal fisting were not associated with (HG)AIN.

**Conclusion:**

GHB use increases the risk for AIN, while duration of cART and anal XTC use are negatively correlated with AIN. Given the high prevalence of AIN in HIV+ MSM, these associations are not helpful to guide a screening program.

## Introduction

During the last two decades the incidence of anal cancer in the HIV-infected population has increased significantly [1,2], especially among HIV+ men who have sex with men (MSM), with incidence rates between 65 and 109 per 100,000 person year[3]. This is much higher than the incidence of cervical cancer in HIV negative women before standard cytological screening was introduced [4], and therefore routine screening for anal premalignant lesions is subject of discussion. In analogy with cervical intraepithelial neoplasia (CIN), premalignant anal lesions are called anal intraepithelial neoplasia (AIN), graded as AIN 1 (low grade (LG)AIN) and AIN 2/3 (high grade (HG)AIN). AIN is present in the majority of HIV+ men who have sex with men (MSM). AIN of any grade is found in 68%-81%, HGAIN in 25-52% [5-7]. For HGAIN, progression rates to anal cancer have been reported to be 14% and 16% among HIV+ MSM, with median follow-up periods of 2 and 5 years respectively [8,9].

Like in CIN, screening and treatment of AIN may be helpful to prevent the development of cancer. The gold standard for AIN detection is high resolution anoscopy (HRA) with biopsies of suspect lesions [10]. Given the high prevalence of HGAIN and low specificity of anal cytology [7], HRA in combination with histopathological examination of lesional biopsies is the preferable first line (HG)AIN screening method. 

Since HRA is time consuming and therefore expensive, not generally available and cumbersome for the patient, it would be useful to identify risk factors for AIN, in order to identify a high-risk population. Previous studies addressing this question used cytological abnormalities as endpoint or cytology as first screening step before HRA [11-18]. The main risk factors identified were receptive anal intercourse (RAI) and HIV infection. The outcomes on other risk factors were conflicting. Two studies performed HRA as primary screening method [19,20]. The presence of high-risk HPV types, in particular HPV16, was a significant risk factor [20]. However, testing for the presence of high-risk HPV types is expensive and not generally available. In addition, these studies did not evaluate other potential risk factors like sexually transmitted infections (STI), anal enemas, sexual habits and drug use, and one of the two only included patients who were HPV 16 positive [19].

In the present study, we investigated a wide range of potential risk factors for AIN, with HRA as primary screening tool. Our goal was to identify factors that might help to identify HIV+ MSM at greatest risk for having premalignant anal (AIN) lesions.

## Methods

### Setting and patients

The study was performed at the HIV and dermatology outpatient clinics of the Academic Medical Center in Amsterdam, the Netherlands. All HIV+ MSM in care over 18 years of age were eligible if they did not have a history of anal cancer or current active inflammatory bowel disease. The study procedures followed were in accordance with the ethical standards of the Helsinki Declaration and the study was approved by the local ethical committee (METC, Academic Medical Center Amsterdam, January 3^rd^, 2008). All patients provided written informed consent.

### Diagnostic procedures and data collection

Screening for AIN was performed by HRA, as described previously [21,22], by a single HRA experienced physician (O.R.). Suspect lesions were biopsied for histopathological analysis, including Ki-67 and p16 immunostaining [23]. A single pathologist (C.v.N.) evaluated all biopsies. Further, all patients were screened for anal chlamydia and gonorrhoea infection.

From the patients’ records, demographic, clinical and laboratory data were collected, including previous STIs, previous anal pathology, year of HIV diagnosis, cART use, nadir CD4 count and current CD4 count and plasma HIV-RNA level, both obtained within the previous 6 months. At the time of HRA, patients were asked for anal complaints and they had to complete a questionnaire addressing number of lifetime sex partners with whom they had receptive anal intercourse, the use of anal enemas, receptive anal fisting (anal insertion of a fist), recreational drug use and anal drug use (anal insertion of recreational drugs). 

### Statistical analysis

Statistical analyses were performed using SPSS software (version 16.0.2 for Windows; SPSS Inc., Chicago, IL, U.S.A.). Diagnosis at HRA was the outcome of interest. In case no suspicious lesions were seen a patient was classified as having no AIN. In case of biopsies, the histopathological diagnosis was used: normal, LGAIN (AIN 1) or HGAIN (AIN 2 or 3). If more than one biopsy was taken, the highest AIN grade was considered as outcome.

Each parameter described above was analysed in univariable logistic regression models with AIN (versus no AIN) or HGAIN (versus no or LGAIN) as independent variable. Parameters with p<0.1 were entered in a stepwise backward multivariable regression model, in which at every step the parameter with the highest p-value larger than 0.05 was removed from the model, until all remaining parameters were significant with p<0.05. 

We finally investigated if these significant parameters would be helpful to identify a population at low risk for having (HG)AIN. Based on the regression equation significant multivariable parameters were combined in one predictor for having (HG)AIN. To estimate the proportion of HIV+ MSM that can be safely excluded from a screening program, the cut-off value of the predictor was calculated for a negative predictive value of 100%.

## Results

### Patients

Between August 2008 and December 2010, 650 HIV+ MSM were screened for eligibility. 191 were not interested, 14 fulfilled the exclusion criteria and 57 had significant comorbidity according to their treating physician. The first 73 patients who underwent HRA were excluded since they participated in a pilot study which has been reported elsewhere [24]. So, for the present study 315 patients underwent HRA. Four patients were excluded from the analysis because the HRA was judged to be of insufficient quality. The median age of the remaining 311 participants was 47 years, and the median duration since HIV diagnosis was 10 years. Eighty-nine percent was using cART, with a median duration of 9 years. Median nadir CD4+ cell count was 200 cells/μl, median current CD4+ cell count was 550 cells/μl and plasma HIV-RNA load was undetectable in 86%. Anal STI screening at inclusion showed in 21/304 (7%) a chlamydia proctitis (of which 4 lymphogranuloma venerum genovar) and gonorrhoea proctitis in 2%. 

Of the patients who answered questions on anal sex, two out of 282 (0,7%) reported that they never had practiced receptive anal intercourse (RAI). Forty-four percent reported RAI with over 50 partners. The majority of participants had used anal enemas prior to RAI, 34% had used over 50 enemas lifetime. Eight patients had been diagnosed with AIN in the past. Baseline characteristics and other details on sexual habits, sexually transmitted diseases, past or present anal pathology, smoking and substance use are given in Table 1.

**Table 1:** Baseline characteristics and univariable logistic regression for AIN (any grade) versus no AIN.

**Characteristic**	**All**	**No AIN**	**AIN**	**OR**	**p**
Age, years	47 (41-55)	47 (42-56)	46 (40-54)	0.98	0.1
**Years since HIV diagnosis**	**10 (5-15)**	**11 (6-15)**	**7 (4-14)**	**0.96**	0.01*
cART	276/311 (89%)	126/136 (93%)	150/175 (86%)	0.48	0.06
**Duration of cART, years**	**9 (2-12)**	**10 (5-12)**	**7 (2-12)**	**0.93**	0.006*
Nadir CD4 count, cells/μl	200 (100-280)	180 (83-268)	200 (110-293)	1.00	0.096
Most recent CD4 count, cells/μl^a^	550 (430-718)	570 (440-750)	540 (410-703)	1.00	0.4
Detectable plasma HIV-RNA load^a^	40/290 (14%)	15/129 (12%)	25/161 (16%)	1.65	0.1
Plasma HIV-RNA viral load, copies/ml^a^	2376 (90-34521)	4619 (61-62778)	1519 (102-80815)	1.00	0.4
Smoking	112/300 (37%)	50/133 (38%)	62/167 (37%)	0.98	0.9
Lifetime no. of RAI partners:					
0-50	153/272 (56%)	72/121 (60%)	81/151 (54%)	NA	NA
>50	119/272 (44%)	49/121 (40%)	70/151 (46%)	1.27	0.3
Lifetime no. of anal enemas prior to sex:					
0	111/271 (41%)	55/120 (26%)	56/151 (37%)	NA	NA
1-50	66/271 (24%)	30/120 (25%)	36/151 (24%)	1.18	0.6
>50	94/271 (34%)	35/120 (29%)	59/151 (39%)	1.66	0.08
Lifetime no. of receptive anal fisting:					
0	205/278 (74%)	87/120 (73%)	118/158 (75%)	NA	NA
1-50	53/278 (19%)	21/120 (18%)	32/158 (20%)	2.12	0.7
>50	20/278 (7%)	12/120 (10%)	8/158 (5%)	0.49	0.1
Lifetime no. of drug use prior/during to sex:					
0	33/257 (13%)	13/103 (13%)	20/154 (13%)	NA	NA
1-50	89/257 (35%)	37/103 (36%)	52/154 (34%)	0.91	0.8
>50	135/257 (53%)	53/103 (51%)	82/154 (53%)	1.01	1
Drug types prior/during sex (ever):					
Poppers	213/235 (91%)	88/98 (90%)	125/137 (91%)	1.18	0.7
Marihuana	129/235 (55%)	56/98 (57%)	73/137 (53%)	0.86	0.6
Amphetamine	49/235 (21) %	15/98 (15%)	34/137 (25%)	1.80	0.08
Metamphetamine	32/235 (14%)	14/98 (14%)	18/137 (13%)	0.90	0.8
Cocaine	103/235 (44%)	39/98 (40%)	64/137 (47%)	1.34	0.3
XTC use	118/235 (50%)	43/98 (44%)	75/137 (55%)	1.50	0.1
**GHB use**	**88/235 (37%)**	**27/98 (28%)**	**61/137 (45%)**	**2.11**	0.009*
Ketamine	55/235 (23%)	19/98 (19%)	36/137 (26%)	1.48	0.2
Lifetime no. of anal drug use:					
0	223/272 (82%)	96/116 (83%)	127/156 (81%)	NA	NA
1-50	42/272 (15%)	14/116 (12%)	28/156 (18%)	1.51	0.2
>50	7/272 (3%)	6/116 (5%)	1/156 (1%)	0.13	0.06
Anal drug use types (ever):					
**Anal XTC**	**12/277 (4%)**	**9/119 (8%)**	**3/158 (2%)**	**0.24**	0.03*
Anal Amphetamine	10/277 (4%)	6/119 (5%)	4/158 (3%)	0.49	0.3
Anal Cocaine	34/277 (12%)	14/119 (12%)	20/158 (13%)	1.09	0.8
Anal GHB	4/277 (1%)	3/119 (3%)	1/158 (1%)	0.25	0.2
Anal Ketamine	14/277 (5%)	5/119 (4%)	9/158 (6%)	1.38	0.6
Current chlamydia/gonnorroea infection	25/304 (8%)	13/134 (10%)	12/170 (7%)	0.71	0.4
Previous STI"s:					
Chlamydia	126/311 (41%)	48/136 (35%)	78/176 (47%)	1.48	0.099
Gonorroea	163/311 (52%)	67/136 (49%)	96/175 (55%)	1.25	0.3
Genital/anal herpes	57/311 (18%)	29/136 (21%)	28/175 (16%)	0.70	0.2
**Anogenital warts**	**130/311 (42%)**	**45/136 (33%)**	**85/175 (49%)**	**1.91**	0.006*
Syphilis	124/311 (40%)	54/136 (40%)	70/175 (40%)	1.01	1
Hepatitis B^b^	141/311 (45%)	69/136 (51%)	72/175 (41%)	0.68	0.09
Hepatitis C^b^	28/311 (9%)	16/136 (12%)	12/175 (7%)	0.56	0.1
Previous anal pathology:					
Hemorrhoids	66/311 (21%)	30/136 (22%)	36/175 (21%)	0.92	0.8
Fissures	25/311 (8 %)	8/136 (6%)	17/175 (10%)	1.72	0.2
Fistulas	18/311 (6%)	8/136 (6%)	10/175 (6%)	0.97	1
Abcess	15/311 (5%)	7/136 (5%)	8/175 (5%)	0.89	0.8
AIN	8/311 (3%)	1/136 (1%)	7/175 (4%)	5.63	0.1

Data are medians (interquartile range) or proportions. Proportions are calculated in relation to the no. of patients for which the specific parameter was applicable and available; ^a^within the previous 6 months; ^b^Active or cleared/treated infection; cART= combination antiretroviral therapy; NA=not applicable; RAI=Receptive Anal Intercourse; GHB=γ-Hydroxybutyric acid; XTC=ecstasy, 3,4-Methylenedioxy-methamphetamine; OR=Odds Ratio; *significant (p<0,05)

### High resolution anoscopy

One hundred and seventy-five (56%) of 311 participants had histopathologically confirmed AIN. Eighty- three patients (27%) had AIN 1, 54 (17 %) AIN 2 and 38 (12%) AIN 3. This means that 92 of 311 patients (30%) had HGAIN. The remaining 136 (44%) did not have suspect lesions on HRA, or AIN was ruled out in biopies taken from suspect lesions.

### Logistic regression

In the univariable analysis (table 1) three factors were significantly associated with a lower risk for AIN: number of years since HIV diagnosis (odds ratio (OR) 0.96 per year (p=0.01), duration of cART treatment with an OR of 0.93 per year (p=0.006) and anal XTC (ecstasy, 3,4-Methylenedioxy-methamphetamine) use ever (OR=0.24; p=0.03). Two factors were associated with a higher risk for AIN: the oral use of GHB (γ-Hydroxybutyric acid) prior to or during sex (OR= 2.11; p=0.009) and a history of anogenital warts (OR= 1.91; p=0.006). Previously reported AIN (n=8) was associated with AIN (OR=5.63), which was not significant (p=0.1), probably because of the low number of cases. 

In multivariable analysis (table 2) three factors remained significant. The duration of cART use (OR of 0.92 per year; 95% confidence interval [CI] 0.87-0.97; p=0.003) and anal XTC use (OR=0.10; 95% CI 0.03-0.45; p=0.002) were negatively associated with AIN and the use of GHB resulted in an increased risk for AIN (OR= 2.60; 95% CI 1.39-4.85; p=0.003). These three parameters were combined into one multivariable predictor for AIN. To reach a negative predictive value of 100%, the cut-off value for the combined predictor was 0.24, with patients scoring below this value being considered as not at risk for AIN. Using this cut-off value, only 3 % of all patients could be rightfully excluded for further screening. 

**Table 2:** Significant predictors of AIN after multivariable logistic regression.

**Characteristic**	**OR**	**95%CI**	**p**
Duration of cART, per year	0.92	0.87-0.97	0.003
GHB use prior/during sex (ever)	2.60	1.39-4.85	0.003
Anal XTC use (ever)	0.10	0.03-0.45	0.002

OR=Odds Ratio; CI=confidence interval; cART=combination antiretroviral therapy; GHB=γ-Hydroxybutyric acid; XTC=ectasy, 3,4 Methylenedioxy-methamphetamine

We repeated logistic regression for HGAIN as compared to no AIN/ LGAIN. No parameters were significantly associated with HGAIN. However, there was a trend towards increased HGAIN risk for anal enema use prior to sex (>50 times ever; p=0.07) and for a history of AIN (p=0.06). 

Nadir CD4 count, current CD4 count, plasma HIV-RNA, previous STIs or anal pathology, lifetime number of RAI partners, and receptive anal fisting were not significantly associated with (HG)AIN. Complaints like diarrhoea, constipation, bloody stool, slimy stool, anal pain, anal itching and erosions could also not be linked to (HG)AIN.

## Discussion

In this study, we analysed a wide range of potential risk factors for AIN and HGAIN in 311 HIV+ MSM. The goal was to identify a high-risk subgroup in order to target future screening programs more efficiently. This is the largest study of its kind in the cART era and the first study in which HRA as primary screening method (without preceding cytology) is combined with both medical history and detailed questionnaires addressing sexual habits and substance use.

 AIN was found in 56% and HGAIN in 30% of 311 participants. In a multivariable analysis, duration of cART and anal XTC use were negatively associated with AIN and GHB significantly increased the risk for AIN. However, a combined multivariable predictor of these three parameters showed that in only 3 % of patients screening could be skipped. No parameters were significantly associated with HGAIN.

The negative association of duration of cART use with AIN is in line with two recent studies in which HRA was also used as primary AIN screening method. The first study showed a lower risk for AIN 2-3 in men using cART: an odds ratio (OR) of 0.28 for receiving cART > 4 years [20]. The second study, which reported odd ratios in relation to *absence* of AIN instead of *presence*, showed a positive association of the use of cART (but not duration of cART) with the absence of AIN, with an OR of 2.28 (p=0.045) [25]. However, this study found an AIN-prevalence among HIV+ MSM of only 16%, which is remarkably low given the current epidemiological data on AIN [3]. Two studies with cytology as primary screening method also reported a relation with cART. The first study showed an OR of 0.18 for receiving cART[17], but in the latter study it was found that in only 4% of patients high grade lesions regressed after initiating cART [14].Other studies do not show any beneficial effect of ART on AIN [11,12,15,16,19]. Likewise, contradictory results are reported on the influence of cART on CIN in HIV positive women [26-28]. In contrast to the influence of cART, we did not find any association between presence of AIN and the CD4 count, suggesting that the benefit of cART is not directly mediated by immune restoration as measured by CD4+ cells. Also, we did not find an association with nadir CD4+ cell count. Some previous studies showed a significant correlation between a low nadir CD4 count and AIN [12,15,20], whereas other studies did not [11,16,19].

Besides cART use, also anal XTC use (ever) was negatively associated with AIN. Anal administration of XTC consists of injecting XTC dissolved in water with a blunt syringe into the anal canal/distal rectum. We did not find any data on mucosal effects of XTC. However, XTC is known to have immune-inhibitory rather than immune stimulating effects [29]. Given the low absolute number of patients reporting anal XTC (n=12), we suspect that the ‘protective’ effect of anal XTC use is most likely a coincident finding. One previous study looked into anal drug use in general, but did not find an association [18].

Although there was a suggestion of increased risk of AIN with a history of (non anal) XTC and amphetamine use, the only recreational drug that showed a significant association with AIN was GHB. GHB is known to have strong disinhibiting behavioural and sexual arousal effects [30]. This might result in more risky sexual behaviour. However, we did not see any influence of GHB use on the number of RAI partners. Therefore, if there is a connection with increased risk behaviour, this might be explained by more unprotected sex and resulting increased exposure to HPV. However, this is speculative since we did not collect data on condom use.

Participants who reported more than 50 anal enemas in their life showed a (non-significant) higher risk for HGAIN. One previous study analysed enema use as a potential risk factor, but did not find an association with AIN [18]. Other studies showed that enema use is significantly associated with lymphogranuloma venereum proctitis, hepatitis B and hepatitis C infection [31-33]. Usually, the enemas contain water, and no irritant substances [31], suggesting that the irrigation procedure itself increases the risk of pathogen transmission, possibly through disruption of the mucosal barrier. Also, sharing of enema equipment with others might play a role. In the case of AIN, enema use might possibly facilitate the transmission of HPV.

Other characteristics, previous STIs, anal pathology, number of RAI partners, anal fisting, anal complaints and smoking were not associated with (HG)AIN. Various other studies investigated one or more of these, but none has been consistently identified as an important risk factor. Older age has been associated with an increased risk of AIN [20], but also the opposite has been found [17]. Although the increase in anal cancer is often attributed to an increased lifespan with HIV, duration since HIV diagnosis is not automatically associated with AIN [20]. Some studies showed a history of anogenital warts as a risk factor, but no other STI seems to be related to AIN [11,18]. Also data on number of sex partners are not conclusive [17,34]. To our knowledge one previous study collected data on anal fisting but, like in this study, did not find an association with AIN [18].

Strong points of our study are the use of HRA with biopsies as primary screening tool, the large number of unselected patients and the wide range of data collected.

Our study has also limitations. Although the physician who performed all HRA’s was sufficiently trained, lesions might have been missed. In that case, the prevalence found in our study is an underestimation. However, the AIN prevalence found is in line with previous data^3^. Further, we did not collect data on condom use. Condom use reduces the risk for anal HPV infection[36], but to our knowledge there are no data on a negative (or positive) correlation between condom use and the presence of AIN. Unsafe sex could also be a confounding factor for both GHB use and anal enemas. Finally, we did not analyse anal HPV. Our goal was to select a target population among HIV+ MSM based on readily available data and questionnaires, without any diagnostic procedure. It is possible that the detection of oncogenic HPV types, the number of HPV types or specific HPV types may contribute to identify those at risk for (HG)AIN. Two recent studies showed that subtyping and load determination of HPV in anal swabs can be useful in predicting (progression to) HGAIN [20,35]. Yet, the vast majority of HIV+ MSM carries multiple oncogenic HPV types [5,7] and HPV analysis is expensive and not generally available. Given the high prevalence of HGAIN, the contribution of HPV analysis needs further investigation, including an evaluation of its cost effectiveness. If only a limited proportion of HIV+ MSM can be excluded from HRA by performing HPV analyses, it might be cost-effective to skip this intermediate step and examine all HIV+ MSM by HRA.

In conclusion, we think that none of the risk factors we found will be useful to target future AIN screening programs in HIV+ MSM. The odds ratios we found were too small to reliably identify those with a low risk of AIN. Given the very high prevalence of HGAIN, screening seems warranted for all HIV+ MSM. 
